# Less Is More? Combined Approaches to Improve Mortality and Morbidity after Aortic Valve Replacement

**DOI:** 10.3390/biomedicines11112989

**Published:** 2023-11-07

**Authors:** Elisa Mikus, Mariafrancesca Fiorentino, Diego Sangiorgi, Simone Calvi, Elena Tenti, Andrea Cavallucci, Elena Tremoli, Alberto Tripodi, Maurizio Pin, Carlo Savini

**Affiliations:** 1 Cardiovascular Department, Maria Cecilia Hospital, GVM Care & Research, 48033 Cotignola, Italy; francescafiorentino@hotmail.it (M.F.); dsangiorgi@gvmnet.it (D.S.); scalvi@gvmnet.it (S.C.); etenti@gvmnet.it (E.T.); acavallucci@gvmnet.it (A.C.); etremoli@gvmnet.it (E.T.); albertotripodi@hotmail.com (A.T.); maurizio.pin10@gmail.com (M.P.); csavini@gvmnet.it (C.S.); 2 Department of Experimental Diagnostic and Surgical Medicine (DIMEC), University of Bologna, 40126 Bologna, Italy

**Keywords:** aortic cross-clamp time, minimally invasive surgery, sutureless aortic valve prosthesis

## Abstract

Background: Nowadays, one of the main goals of aortic valve surgery is to reduce the biological impact, mortality, and complications. It is well-known that long operative times in terms of the extracorporeal circulation, but above all, of the aortic cross-clamp time (ACC), represent a risk factor for mortality in patients undergoing cardiac surgery. In order to shorten the aortic cross-clamp time, many technological improvements, such as sutureless prostheses, have been introduced, but their actual effectiveness has not been proven yet. The aim of this study was to assess the 30-day outcomes of patients undergoing aortic valve replacement surgery, focusing on the ACC length. Methods: All 3139 patients undergoing aortic valve replacement between January 2013 and July 2022 at our institution were enrolled. The data were retrospectively collected and the baseline characteristics and intraoperative variables were recorded. In order to adjust the results according to the differences in the baseline characteristics, propensity score matching was performed and four groups of 351 patients were obtained based on the first, second, third, and fourth quartile of the ACC time. Results: The patient population included 132 redo surgeries (9.4%) and 61 cases of active endocarditis (4.3%), with an overall median EuroSCORE II of 1.8 (IQR 1.2–3.1). An increase across the groups was observed in terms of the acute kidney failure (*p* < 0.001) incidence, the number of blood transfusions (*p* = 0.022), prolonged hospital stays (*p* < 0.001), the and respiratory failure (*p* < 0.001) incidence. A *p* of < 0.1 was found for the 30-day mortality (*p* = 0.079). The predictors of an early 30-day mortality were standard full sternotomy (OR 2.48, 95% CI 1.14–5.40, *p* = 0.022), EuroSCORE II (OR 1.10, 95% CI 1.05–1.16, *p* < 0.001), and a trend for a longer ACC time (Q4 vs. Q1: OR 2.62, 95% CI 0.89–7.68, *p* = 0.080). Conclusions: Shortening the operative times resulted in marked improvements of the patients’ outcomes. The combined use of minimally invasive approaches and sutureless aortic valve prostheses allows for a lower 30-day events rate. New technologies should be assessed to obtain the best results with the least risk.

## 1. Introduction

In the last decade, the burden of aortic valve disease has received growing attention, especially for aortic valve stenosis due to its frequency in the elderly. Indeed, the prevalence of aortic valve stenosis, characterized by leaflet stiffening and thickening, increases with age, affecting 3.4% of the elderly population >75 years [1,2]. Considering the potential increase in the incidence of aortic valve diseases, it is necessary to define the gold standard for its surgical treatment and to the identify risk factors for mortality and adverse events occurrence. Cardiac surgery for aortic valve replacement has evolved both in terms of techniques and technological supports, with the aim to simplify the procedure and to reduce patients’ surgical trauma. In this scenario, since 1996, minimally invasive surgical accesses have been proposed, when Cosgrove and Sabik reported the first parasternal approach for mini AVR [3]. Actually, ministernotomy and minithoracotomy are the most popular minimally invasive approaches, with excellent results [4,5]. However, for several years, the use of minimally invasive approaches caused an increase in extracorporeal circulation and cross-clamp times, thus enhancing the biological aggressiveness of the surgery [6]. Indeed, in the literature, different authors agree on the fact that, in order to obtain an optimal surgical intervention with a lower 30-day mortality and morbidity, it is mandatory to reduce the ACC times [7,8,9]. At the same time, the availability of sutureless aortic prostheses allowed for the reduction in the length of ACC times, even when using minimally invasive approaches [10,11,12].

The aim of this study is to define the real impact of the ACC time on patients’ minor and major outcomes after aortic valve replacement surgery. We decided to analyze a real-world patients’ population, as homogeneous as possible both in terms of type of surgical procedure and in terms of pre-operative characteristics and risk factors. The role of minimally invasive approaches and sutureless prostheses will be assessed.

## 2. Materials and Methods

Demographic and major baseline characteristics data (age, sex, body mass index, creatinine clearance, preoperative condition, cardiovascular risk factors, functional status, and left ventricular ejection fraction, EuroSCORE II) were collected and stored, together with intraoperative and short-term outcomes. Echocardiography was performed pre- and postoperatively. Clinical follow-up was performed at 30 days, either with telephone interviews or patient visits.

### 2.1. Study Population

This is a monocentric, retrospective, single-center study including 3139 consecutive adult patients (aged > 18 years) with severe aortic valve disease (stenosis or regurgitation) scheduled for a surgical aortic valve replacement from January 2013 to July 2022. No sample size calculation was performed, but all available patients were included in our study.

Informed consent was obtained from all subjects involved in the study; the data were gathered, starting from clinical charts and collected in a specific registry, and every possible effort has been made to reduce missing information. Since in our database’s missing values are referred to as missingness completely at random and arising directly from gaps in clinical charts, only complete cases were analyzed. In order to obtain homogeneous subgroups of patients in relation to the ACC time, the patient population was divided into 4 groups based on the quartiles of the operative ACC time (short, medium-short, medium-long, long ACC time): the first group had a median ACC time equal to 32 min (interquartile range 13–38); the second group equal to 45 min (39–51); the third group equal to 58 min (52–66) and the fourth group equal to 78 min (67–656). Because of the retrospective nature of the study, in order to reduce the selection bias, and therefore to balance the 4 groups, propensity score matching was performed and we obtained 4 groups of 351 patients each (Table 1). Only patients with non-missing values in the baseline covariates were retained for matching. The resulting 351 patients per group are those with non-missing values selected using the propensity score matching procedure.

The surgical technique adopted for aortic valve replacement using traditional median sternotomy or minimally invasive approaches (minithoracotomy or ministernotomy) has been previously described [13].

### 2.2. Endpoint

The primary end point of the study was the 30-day mortality according to the ACC time category. The secondary endpoint was the incidence of postoperative adverse events in the 4 groups. In addition, the role of minimally invasive approaches and sutureless prostheses was assessed.

### 2.3. Statistical Analysis

Patients were divided into 4 groups according to the quartiles of the cross-clamp time. After checking for normality with the Shapiro–Wilk test, the continuous variables were reported as the median and interquartile range (IQR) and compared with the Kruskal–Wallis test. The categorical variables were reported as the absolute number and frequencies and compared with the chi-squared test or Fisher exact test as appropriate. In order to minimize the selection bias and balance the 4 groups, ordinal logistic regression (packages ologit and predict) considering all baseline characteristics was applied. Only the patients with non-missing values in the baseline covariates were retained for matching; the groups were matched according to stratification in quintiles of the probabilities obtained from the multivariable model. The absolute standardized mean differences (ASMD) were reported in order to assess the balancing across groups; to consider the worst unbalanced comparison, the maximum pairwise ASMD was reported; the variables with an ASMD < 0.2 were considered as balanced [14]. For the categorical outcomes, the Cochran–Armitage test for trend was reported, multivariable logistic regression models were performed, the model discrimination was assessed using the c-statistic, while the model calibration was assessed using the Hosmer–Lemeshow test [15]. When necessary to avoid overfitting, model selection based on the AIC minimization criteria was applied. For the continuous outcomes, Cuzick’s test for trend was reported [16]. A generalized linear model with a log link function and gamma distribution for the continuous data and a negative binomial for the count variables were performed. The Anscombe residuals were analyzed for normality, marginal effect plots were reported, and the covariates that were still statistically different at a *p* < 0.1 level after PSM were included in all the models. All analyses were performed with STATA 18.0 SE (StataCorp LLC, College Station, TX, USA); *p*-values < 0.05 were considered statistically significant.

## 3. Results

### 3.1. Baseline Characteristics

The baseline characteristics of the whole patients’ population, divided into four groups according to the quartiles of the ACC time, are reported in Supplementary Table S1. After propensity score (PS) matching, the cohorts were equally matched. All the baseline patient characteristics were well-balanced (standardized mean difference < 0.2) including the patients’ age and EuroSCORE II, even if a statistically significant difference remained for these variables (Table 1).

The surgical data and in-hospital outcomes according to the quartiles of the ACC time are reported in Table 2. Aortic valve replacement surgeries were performed through minimally invasive accesses in 73.1% of cases, using both ministernotomy (52.9%) or minithoracotomy (20.2%). The percentage, however, of minimally invasive approaches, compared to the traditional full sternotomy in the four groups was similar (*p* = 0.061). Probably, the shorter cross-clamp time of the patients in group 1 (Q1) was due to the greater number of implanted sutureless Perceval aortic bioprostheses (Corcym SRL, Milan, Italy) (*p* for trend < 0.001). This variable is the only one capable of justifying the different duration of the ACC time among groups. No other specific reasons for the differences in the ACC time were identified.

### 3.2. Postoperative Outcomes

The comparison between the four groups showed an increase in terms of the acute kidney failure (defined as eGFR < 60 mL/min) incidence (*p* < 0.001), the number of blood transfusions (*p* = 0.022), a prolonged hospital stay of more than 21 days (*p* < 0.001), as well as the respiratory failure (*p* < 0.001) incidence across groups. Moreover, a *p* < 0.1 was found for the 30-day mortality (*p* = 0.079) and intra-aortic balloon pump (IABP) implantation (*p* = 0.062) among groups. Concerning other postoperative variables, a progressively worsening trend of the postoperative outcomes was observed from Q1 to Q4. This was evident for the in-hospital mortality, re-sternotomy for bleeding, sepsis and intensive care unit stay with a *p* < 0.2. The only exception was the incidence of sternal diastasis, which resulted in being more frequent in Q1. The in-hospital mortality of the patients assigned to Q1 was 1.4% and this value was more than doubled in the Q4 group, even if the difference did not reach statistical significance (3.1%; *p* = 0.137), probably due to the relatively low number of events.

**Figure 1:**
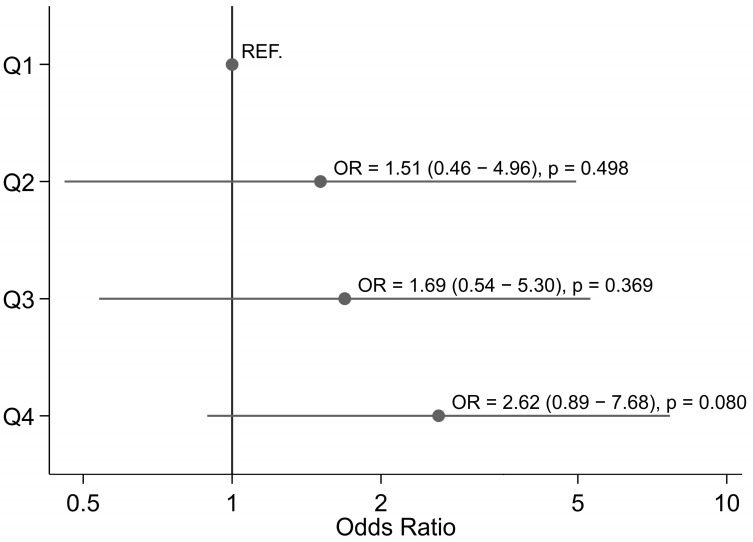
Forest plot for death at 30 days according to quartiles of ACC time. Model adjusted for covariates still significant after propensity score matching (age, EuroSCORE II, smoke, hypertension, full sternotomy).

The predictors of the 30-day mortality are reported in Figure 1. From the multivariable analysis, the following factors were identified as significant: standard full sternotomy (OR 2.48, 95% CI 1.14–5.40, *p* = 0.022) and EuroSCORE II (OR 1.10, 95% CI 1.05–1.16, *p* < 0.001). Moreover, a trend for a longer cross-clamp time (Q4 vs. Q1: OR 2.62, 95% CI 0.89–7.68, *p* = 0.080) was found (Figure 1). The area under curve was 0.729.

**Figure 2:**
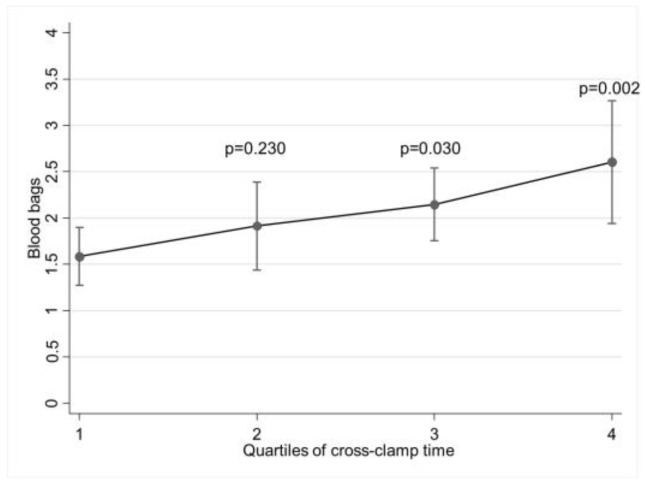
Marginal effects plot for red blood transfusion according to quartiles of ACC time. Model adjusted for covariates still significant after propensity score matching (age, EuroSCORE II, smoke, hypertension, full sternotomy).

From the multivariable analysis, the number of red blood cell (RBC) units used in Q3 (*p* = 0.030) and Q4 (*p* = 0.002) resulted as being higher compared to Q1 (Figure 2), whereas the difference in the ventilation time did not reach statistical significance.

**Figure 3:**
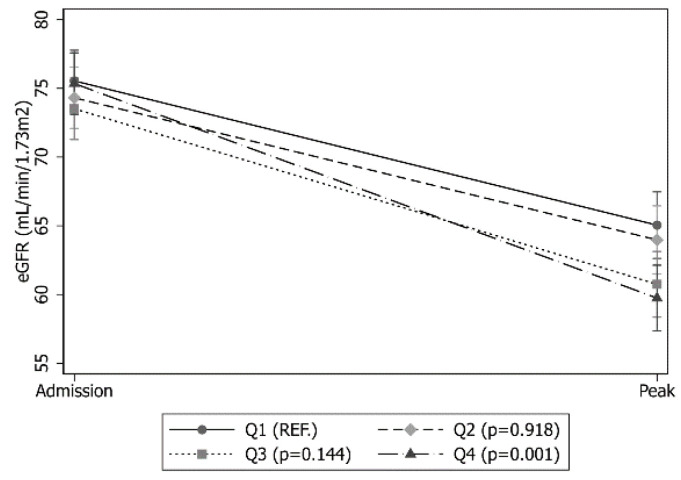
Marginal effects plot for estimated glomerular filtration rate according to quartiles of ACC time. Model adjusted for covariates still significant after propensity score matching (age, EuroSCORE II, smoke, hypertension, full sternotomy).

The estimated glomerular filtration rate (eGFR) showed a decrease equal to 9.7, 12.2, 13.5, and 16.4 from Q1 to Q4, respectively, with a statistical significance only for Q4 (*p* = 0.001) (Figure 3). The incidence of the eGFR < 60 showed a strong trend across the four quartiles (*p* < 0.001).

## 4. Discussion

To the best of our knowledge, this is the first study that analyzes a homogeneous population of patients, both considering the type of intervention (isolated aortic valve replacement) and the clinical characteristics, with the aim of assessing the potential effect of the ACC time on the patients’ outcomes. This PS-matched study considered the early outcomes of patients undergoing surgical aortic valve replacement either with full sternotomy or with mini-invasive approaches, both using sutureless and sutured prostheses in a real-world cohort.

Our data showed that

- Patients with a longer ACC time had a higher incidence of acute kidney failure, number of blood transfusions, prolonged hospital stay (more than 21 days), as well as respiratory failure;
- The predictors of early 30-day mortality were standard full sternotomy, EuroSCORE II, and a trend for a longer aortic cross-clamp time

Over the years, cardiac surgery has evolved both in terms of techniques and technological supports, with the aim to decrease the invasiveness and to subsequently reduce the recovery time for patients as much as possible. In this scenario, different minimally invasive approaches for aortic valve surgery have been introduced. Currently, the two most popular approaches to mini surgical aortic valve replacement (SAVR) include mini-sternotomy and mini-thoracotomy. Of note, minimally invasive approaches may be more technically challenging, especially the mini-thoracotomy, due to its relatively restricted operative field, resulting in longer aortic cross-clamp times [6]. Can we actually define a surgical procedure to be minimally invasive when this causes an increase in the extracorporeal circulation times, increasing the biological surgical invasiveness? What is the impact of the aortic cross-clamp time on the patients’ outcomes? Back in 1985, Blackstone and Kirklin [17] showed that the ACC time is an independent risk factor for mortality. Later on, many authors confirmed this finding [7,8,9]. Flameng et al. [8], in 1996, within a multivariable model, identified the ACC time as an independent risk factor for early (90-day) mortality, with a mortality risk increase of 1.8% per one-minute increase in the ACC time, in patients affected by valvular and coronary artery disease. More than ten years ago, in 2012, Ranucci [7] published a popular article about the role of aortic cross clamp time on outcomes of patients undergoing isolated aortic valve surgery. One of the main findings of the study was that the aortic cross-clamp time is an independent determinant of severe cardiac morbidity in patients undergoing aortic valve replacement, but the ACC time could not be identified as a predictor of operative mortality. In his article, the odds ratio for mortality (1.5% increase per 1 min increase in ACC time) was consistent with the data published by Flameng. Based on these data and with the aim of reducing ACC times, especially in minimally invasive approaches, sutureless aortic valve prostheses were introduced. Sutureless aortic valves are particularly advantageous as they obviate the need of using stiches to fix the valve, hence making the procedure faster. Actually, the Corcym Perceval aortic bioprosthesis (Corcym SRL, Milan, Italy), in particular, is the most widely used sutureless aortic valve around the globe. The Perceval S bioprosthesis is constructed from bovine pericardium fixed in a metal cage made up of an alloy of nickel and titanium, known as nitinol. The nitinol cage can be compressed for the implantation and then released to reach its final diameter. The inflow ring of the valve has three loops corresponding to each sinus of the valve, through which the sutures are passed as a guide to aid the prosthetic positioning in the native annulus, and are then removed. Since their first use in aortic valve surgery, several studies assessed the potential benefit of sutureless prostheses. Thanks to the availability of sutureless prostheses, it has been possible to shorten the aortic cross-clamp time even using minimally invasive approaches. Moreover, in the case of small and fragile aortic annulus, sutureless prostheses can be used to prevent bleeding from the root and limit potential aortic complications. It should be noted, however, that in the case of sutureless prosthesis use, a higher rate of pacemaker implantation has been reported [18]. Although, in our patient population, in group 1 (Q1), compared to the others, there is a higher percentage of patients in which sutureless prostheses were used, the pacemaker implantation rate was not significantly different from that of the other groups, where less sutureless prostheses were implanted (*p* for trend 0.687) [19]. However, the superiority of sutureless valves over sutured prostheses has not been proven as of yet [20,21,22]. In 2022, Beretta et al. published a PS-matched study [21], in which they assessed the early outcomes of patients undergoing sutureless and rapid deployment aortic valve replacement (SURD-AVR) versus sutured aortic valve replacement (s-AVR) in an all comers real-life registry cohort. The results showed that (i) SURD-AVR was associated with shorter operative times and an increased rate of less invasive approaches, (ii) while the in-hospital mortality was low and similar between SURD-AVR and s-AVR, the occurrence of stroke was higher in patients who underwent SURD-AVR in the first study period, and (iii) when compared with s-AVR, SURD-AVR showed a reduced incidence of postoperative atrial fibrillation, low cardiac output syndrome, and mild AR, but a higher rate of PM implantation.

Another key point to discuss is that, even if a quick implantation makes the ACC time, cardiopulmonary bypass time, and overall procedure time significantly shorter, is this reduction clinically relevant in a straightforward case of isolated aortic valve replacement? To answer this question, we decided to set up our study with a different methodology from that implemented by Ranucci and others. In fact, we did not analyze the ACC time as an independent risk factor for morbidity and mortality, but we recorded all the patients’ clinical postoperative outcomes and we divided the patients according to the ACC times. Basically, we decided to analyze a real-world patient population, as homogeneous as possible, both in terms of the type of surgical procedure and in terms of the pre-operative characteristics and risk factors. All 3139 consecutive adult patients scheduled for isolated surgical aortic valve replacement were enrolled. The median and the quartile of the ACC time was obtained and the patients were divided into four groups based on the first, second, third, and fourth quartile of the ACC time. To balance the four groups, propensity score matching was performed and we obtained four groups of 351 patients each. All groups are similar between each other and they did not show any pre-operative characteristic that could predict a longer or shorter duration of the ACC time. The results obtained answered our former question: with the same pre-operative risk factors, patients’ outcomes worsen with an increase in the ACC time. Our data indicate that longer ACC times are associated with an increase in the acute kidney failure incidence, number of blood transfusions, a prolonged hospital stay (more than 21 days), as well as respiratory failure incidence. Furthermore, a trend in a higher 30-day mortality rate is also observed even if this is to be confirmed in a larger sample, since our result does not reach statistical significance (*p* < 0.1).

It is also important to underline that the minimally invasive approaches, performed both with ministernotomy or right anterior minithoracotomy, appear to have a protective role against mortality. This is probably related to the fact that, in minimally invasive approaches, where the surgical space is limited and visibility is often worse than in conventional full sternotomy, sutureless prostheses have been used more frequently, thus reducing the ACC time. In addition, a similar result was observed in a previous paper published in 2021, focused on aortic valve surgery in obese patients [23] and confirmed by Abdelaal SA et al. [24]. Moreover, similar results were published last year in a systematic review and meta-analysis published by El-Andari [25]; this study found that the in-hospital mortality rates were higher using full sternotomy than ministernotomy (*p* = 0.02) and that the 30-day mortality was higher using full sternotomy compared to right anterior thoracotomy (*p* = 0.006). Furthermore, both mini-thoracotomy and mini-sternotomy have been the first choice of approach in isolated aortic valve surgery for many years, while full sternotomy is used in only in a small percentage of patients (26.9%). Of course, minimally invasive surgery requires training and practice to optimize the results. On the other hand, in our institution, a standard protocol for patients’ management is applied, regardless of the surgical technique used. This might explain the lack of differences in the ventilation times, and the intensive care unit and in-hospital length of stay between groups. Interestingly, the percentage of long in-hospital stays was, however, greater in the Q3 and Q4 groups (*p* < 0.001) resulting in an increased use of resources and costs per patient.

## 5. Limitations

This study has several limitations. First, the study is not randomized and all variables are retrospectively collected. Second, the choice of surgical approach and aortic valve prosthesis was not defined on the basis of specific criteria but was left to the surgeon’s decision. In addition, this study represents the experience of a single surgical center without a formal sample size calculation. Despite the above-mentioned limitations, we believe that this work is relevant, as it both describes a real-world patient population and underlines the importance of a short aortic cross-clamp time, even in a straightforward case of isolated aortic valve replacement.

## 6. Conclusions

To date, it is well-known that the ACC time in cases of surgical aortic valve replacement represents a risk factor for mortality and/or morbidity. In our study, the reduction of the aortic cross-clamping time has been shown to be associated with a lower incidence of acute kidney failure, number of blood transfusions, prolonged hospital stays (more than 21 days), as well as respiratory failure. The predictors of an early 30-day mortality resulted as being the standard full sternotomy and EuroSCORE II. We can hypothesize that the use of sutureless prostheses, which can compensate for the increased surgical times of a minimally invasive approach, allowed us to reduce the ACC times, with a consequent improvement of patients’ outcomes after aortic valve surgery, but this is not proven. On the other hand, every available new technology that can reduce the ACC time must be used in order to obtain the best results with minor risks.

## Figures and Tables

**Table 1:** Baseline demographic and clinical characteristics (after propensity score matching).

		Aortic Cross-Clamp Time					
	Overall	Q1 (13–38)	Q2 (39–51)	Q3 (52–66)	Q4 (67–656)	*p*	ASMD
N	1404	351	351	351	351		
Age, median (IQR)	75 (69–79.5)	76 (70–80)	75 (68–79)	76 (70–80)	74 (67–79)	0.027	0.186
Female gender (n, %)	615 (43.8)	163 (46.4)	156 (44.4)	157 (44.7)	139 (39.6)	0.297	0.138
Height, cm, median (IQR)	166 (160–172)	165 (160–172)	165 (160–172)	166 (160–172)	168 (160–172)	0.443	0.101
Weight, kg, median (IQR)	75 (67–85)	75 (66–85)	77 (67–87)	74 (65–85)	77 (68–85)	0.089	0.167
BMI, median (IQR)	27.3 (24.4–30.1)	27.2 (24.2–29.8)	27.7 (24.7–30.9)	27.0 (24.2–29.8)	27.3 (24.6–30.4)	0.110	0.167
Hypertension (n, %)	1075 (76.6)	255 (72.6)	273 (77.8)	283 (80.6)	264 (75.2)	0.076	0.189
Diabetes (n, %)	352 (25.1)	90 (25.6)	96 (27.4)	88 (25.1)	78 (22.2)	0.467	0.119
Dyslipidemia (n, %)	825 (58.8)	205 (58.4)	212 (60.4)	200 (57.0)	208 (59.3)	0.825	0.069
Smoke (n, %)	567 (40.4)	129 (36.8)	135 (38.5)	143 (40.7)	160 (45.6)	0.093	0.180
Preoperative atrial fibrillation (n, %)	131 (9.3)	33 (9.4)	23 (6.6)	38 (10.8)	37 (10.5)	0.192	0.152
Pacemaker (n, %)	13 (0.9)	1 (0.3)	4 (1.1)	4 (1.1)	4 (1.1)	0.553	0.102
NYHA class (n, %)						0.485	0.164
- I	217 (15.5)	58 (16.5)	46 (13.1)	52 (14.8)	61 (17.4)		
- II	715 (50.9)	182 (51.9)	178 (50.7)	170 (48.4)	185 (52.7)		
- III	450 (32.1)	104 (29.6)	120 (34.2)	125 (35.6)	101 (28.8)		
- IV	22 (1.6)	7 (2.0)	7 (2.0)	4 (1.1)	4 (1.1)		
CCS class (n, %)						0.885	0.150
- 0	952 (67.8)	246 (70.1)	227 (64.7)	236 (67.2)	243 (69.2)		
- 1	293 (20.9)	70 (19.9)	80 (22.8)	73 (20.8)	70 (19.9)		
- 2	133 (9.5)	31 (8.8)	35 (10.0)	35 (10.0)	32 (9.1)		
- 3	22 (1.6)	3 (0.9)	8 (2.3)	5 (1.4)	6 (1.7)		
- 4	4 (0.3)	1 (0.3)	1 (0.3)	2 (0.6)	0 (0.0)		
Bicuspid aortic valve (n, %)	127 (9.0)	32 (9.1)	33 (9.4)	24 (6.8)	38 (10.8)	0.322	0.141
Aortic stenosis (n, %)	1056 (75.2)	260 (74.1)	277 (78.9)	265 (75.5)	254 (72.4)	0.224	0.153
Aortic regurgitation (n, %)	188 (13.4)	60 (17.1)	40 (11.4)	46 (13.1)	42 (12.0)	0.112	0.163
LVEF %, median (IQR)	60 (55–65)	60 (55–65)	60 (55–65)	60 (55–65)	60 (55–65)	0.678	0.093
Active endocarditis (n, %)	61 (4.3)	14 (4.0)	10 (2.8)	18 (5.1)	19 (5.4)	0.323	0.129
Previous stroke (n, %)	44 (3.1)	11 (3.1)	14 (4.0)	10 (2.8)	9 (2.6)	0.726	0.080
Previous TIA (n, %)	31 (2.2)	7 (2.0)	12 (3.4)	6 (1.7)	6 (1.7)	0.352	0.108
Significant carotid artery disease (n, %)	8 (0.6)	3 (0.9)	1 (0.3)	0 (0.0)	4 (1.1)	0.170	0.152
Creatinine, mg/dL, median (IQR)	0.96 (0.80–1.13)	0.95 (0.79–1.11)	0.96 (0.80–1.14)	1.00 (0.81–1.15)	0.94 (0.80–1.11)	0.271	0.041
CKD stage (n, %)						0.400	0.145
- 1	329 (23.4)	84 (23.9)	82 (23.4)	75 (21.4)	88 (25.1)		
- 2	709 (50.5)	174 (49.6)	182 (51.9)	168 (47.9)	185 (52.7)		
- 3	332 (23.6)	87 (24.8)	75 (21.4)	101 (28.8)	69 (19.7)		
- 4	25 (1.8)	4 (1.1)	9 (2.6)	6 (1.7)	6 (1.7)		
- 5	9 (0.6)	2 (0.6)	3 (0.9)	1 (0.3)	3 (0.9)		
Chronic lung disease (n, %)	130 (9.3)	36 (10.3)	25 (7.1)	36 (10.3)	33 (9.4)	0.432	0.111
Previous cardiac surgery (n, %)	132 (9.4)	37 (10.5)	29 (8.3)	33 (9.4)	33 (9.4)	0.784	0.078
EuroSCORE logistic, median (IQR)	6.19 (3.99–9.21)	6.31 (4.52–9.37)	5.92 (3.99–8.90)	6.32 (4.26–10.38)	5.48 (3.48–8.88)	0.006	0.143
EuroSCORE II, median (IQR)	1.80 (1.19–3.05)	1.89 (1.19–3.20)	1.72 (1.19–2.92)	1.92 (1.29–3.16)	1.69 (1.12–2.82)	0.025	0.066
Previous dialisys (n, %)	6 (0.4)	1 (0.3)	2 (0.6)	0 (0.0)	3 (0.9)	0.341	0.131
Urgency (n, %)	170 (12.1)	42 (12.0)	44 (12.5)	36 (10.3)	48 (13.7)	0.571	0.105
CPB minutes, median (IQR)	63.0 (49.0–80.0)	42.0 (37.0–46.0)	56.0 (52.0–60.0)	70.0 (66.0–75.0)	91.5 (84.0–103.0)	<0.001	/

ASMD: absolute standard mean difference. BMI: body mass index. CCS: The Canadian Cardiovascular Society Angina Score. CKD: chronic kidney disease. CPB: cardiopulmonary bypass. EuroSCORE: European system for cardiac operative risk evaluation. LVEF: left ventricle ejection fraction. NYHA: New York Heart Association. TIA: transient ischemic attack.

**Table 2:** Outcomes and health resources consumption.

		Aortic Cross Clamp Time				
	Overall	Q1	Q2	Q3	Q4	*p* for Trend *
N	1404	351	351	351	351	
Atrial fibrillation (n, %)	445 (31.7)	101 (28.8)	122 (34.8)	108 (30.8)	114 (32.5)	0.521
Pacemaker (n, %)	46 (3.3)	14 (4.0)	8 (2.3)	8 (2.3)	16 (4.6)	0.687
Re-open (n, %)	43 (3.1)	10 (2.8)	7 (2.0)	11 (3.1)	15 (4.3)	0.188
Tamponade (n, %)	11 (0.8)	3 (0.9)	2 (0.6)	3 (0.9)	3 (0.9)	0.892
Cardiac arrest (n, %)	9 (0.6)	2 (0.6)	2 (0.6)	2 (0.6)	3 (0.9)	0.654
Re-intubation (n, %)	39 (2.8)	9 (2.6)	9 (2.6)	10 (2.8)	11 (3.1)	0.611
Tracheostomy (n, %)	15 (1.1)	4 (1.1)	3 (0.9)	4 (1.1)	4 (1.1)	0.908
Sepsis (n, %)	30 (2.1)	4 (1.1)	11 (3.1)	10 (2.8)	5 (1.4)	0.869
Multi organ failure (n, %)	13 (0.9)	2 (0.6)	2 (0.6)	4 (1.1)	5 (1.4)	0.170
Inotropic drug (n, %)	56 (4.0)	10 (2.8)	14 (4.0)	18 (5.1)	14 (4.0)	0.329
Delirium (n, %)	84 (6.0)	20 (5.7)	13 (3.7)	28 (8.0)	23 (6.6)	0.227
Stroke (n, %)	15 (1.1)	2 (0.6)	4 (1.1)	5 (1.4)	4 (1.1)	0.416
Dialysis (n, %)	15 (1.1)	4 (1.1)	4 (1.1)	3 (0.9)	4 (1.1)	0.908
IABP (n, %)	13 (0.9)	1 (0.3)	3 (0.9)	3 (0.9)	6 (1.7)	0.062
ECMO (n, %)	4 (0.3)	0 (0.0)	1 (0.3)	2 (0.6)	1 (0.3)	0.370
Diastasis revision (n, %)	4 (0.3)	2 (0.6)	1 (0.3)	1 (0.3)	0 (0.0)	0.179
In-hospital death (n, %)	30 (2.1)	5 (1.4)	7 (2.0)	7 (2.0)	11 (3.1)	0.137
Death at 30 days (n, %)	32 (2.3)	5 (1.4)	7 (2.0)	8 (2.3)	12 (3.4)	0.079
eGFR < 60 incidence (n, %)	276 (19.7)	51 (14.5)	53 (15.1)	73 (20.8)	99 (28.2)	<0.001
Transfusion (n, %)	790 (56.3)	187 (53.3)	194 (55.3)	201 (57.3)	208 (59.3)	0.092
- Blood, mean (SD)	2.0 (4.6)	1.7 (3.3)	1.8 (4.1)	2.1 (3.7)	2.5 (6.6)	0.022
- Plasma/platelets (n, %)	64 (4.6)	9 (2.6)	12 (3.4)	18 (5.1)	25 (7.1)	0.002
Ventilation hours, mean (SD)	12.2 (52.5)	11.0 (55.3)	9.5 (20.8)	13.1 (41.0)	15.3 (76.6)	<0.001
>24 h (n, %)	41 (2.9)	8 (2.3)	9 (2.6)	13 (3.7)	11 (3.1)	0.357
>48 h (n, %)	25 (1.8)	5 (1.4)	6 (1.7)	8 (2.3)	6 (1.7)	0.652
>72 h (n, %)	20 (1.4)	3 (0.9)	5 (1.4)	7 (2.0)	5 (1.4)	0.420
ICU days, mean (SD)	2.7 (2.3)	2.7 (2.4)	2.5 (1.9)	2.7 (2.2)	2.8 (2.6)	0.363
- ICU days, 3+ (n, %)	369 (26.3)	91 (25.9)	80 (22.8)	93 (26.5)	105 (29.9)	0.130
Length of stay (days), mean (SD)	9.9 (4.0)	10.1 (4.1)	9.6 (3.9)	10.1 (4.0)	9.9 (4.1)	0.259
- Total days, 21+ (n, %)	170 (12.1)	27 (7.7)	38 (10.8)	50 (14.2)	55 (15.7)	<0.001
Sutureless (n, %)	328 (23.4)	147 (41.9)	85 (24.2)	62 (17.7)	34 (9.7)	<0.001
Mini-approaches (n, %)	1027 (73.1)	245 (69.8)	269 (76.6)	246 (70.1)	267 (76.1)	0.247
- Ministernotomy (n, %)	743 (52.9)	164 (46.7)	194 (55.3)	189 (53.8)	196 (55.8)	
- Minithoracotomy (n, %)	284 (20.2)	81 (23.1)	75 (21.4)	57 (16.2)	71 (20.2)	

* For categorical outcomes, the Cochran–Armitage test for trend was reported. For continuous outcomes Cuzick’s test for trend was reported [16]. IABP: intra-aortic balloon pump. ECMO: extracorporeal membrane oxygenation. eGFR: estimated glomerular filtration rate. ICU: intensive care unit.

## Data Availability

The data presented in this study are available on request from the corresponding author. The data are not publicly available due to data protection directive 95/46/EC.

## Supplementary Materials

The following supporting information can be downloaded at: https://www.mdpi.com/article/10.3390/biomedicines11112989/s1, Table S1: Baseline demographic and clinical characteristics (pre-propensity score matching).
