# Supplementary material for: Less Is More? Combined Approaches to Improve Mortality and Morbidity after Aortic Valve Replacement
Source: Biomedicines. 2023 Nov 7;11(11):2989. doi: 10.3390/biomedicines11112989 (PMC10669498; doi:10.3390/biomedicines11112989)
Supplement: Supplementary file 1 [file biomedicines-11-02989-s001.zip › biomedicines-2634352-supplementary.pdf]

Supplementary Materials

Table S1. Baseline demographic and clinical characteristics (pre propensity score matching).

	Clamp time						
	**Overall**	**Q1**	**Q2**	**Q3**	**Q4**	**p**	**SMD**
N	3,139	796	805	800	738		
Age, median (IQR)	75 (69-80)	76 (71-80)	75 (69-80)	75 (69-79)	73 (64-78)	<0.001	**0.390**
Female gender (n, %)	1,453 (46.3)	433 (54.4)	405 (50.3)	358 (44.8)	257 (34.8)	<0.001	**0.401**
Height, cm, median (IQR)	165 (160-172)	164 (159-170)	165 (160-172)	166 (160-173)	169 (160-173)	<0.001	**0.383**
Weight, kg, median (IQR)	75 (65-85)	73 (63-81)	75 (65-84)	75 (66-85)	77 (70-86)	<0.001	**0.396**
BMI, median (IQR)	27.0 (24.3-30.0)	26.8 (23.8-29.4)	27.0 (24.3-30.1)	27.0 (24.5-29.8)	27.4 (24.6-30.5)	0.002	0.199
Hypertension (n, %)	2,406 (76.6)	605 (76.0)	624 (77.5)	629 (78.6)	548 (74.3)	0.201	0.103
Diabetes (n, %)	606 (19.3)	161 (20.2)	188 (23.4)	142 (17.8)	115 (15.6)	0.001	0.197
Dyslipidemia (n, %)	1,815 (57.8)	476 (59.8)	474 (58.9)	450 (56.3)	415 (56.2)	0.359	0.072
Smoke (n, %)	1,265 (40.3)	304 (38.2)	297 (36.9)	335 (41.9)	329 (44.6)	0.008	0.157
Preoperative Atrial Fibrillation							
(n, %)	289 (9.2)	72 (9.0)	53 (6.6)	85 (10.6)	79 (10.7)	0.014	0.147
Pacemaker (n, %)	27 (0.9)	3 (0.4)	7 (0.9)	8 (1.0)	9 (1.2)	0.323	0.095
NYHA class (n, %)						0.437	0.131
- I	486 (15.5)	123 (15.5)	129 (16.0)	107 (13.4)	127 (17.2)		
- II	1,498 (47.7)	389 (48.9)	388 (48.2)	370 (46.3)	351 (47.6)		

- III	1,077 (34.3)	265 (33.3)	272 (33.8)	300 (37.5)	240 (32.5)		
- IV	65 (2.1)	17 (2.1)	13 (1.6)	18 (2.3)	17 (2.3)		
- missing	13 (0.4)	2 (0.3)	3 (0.4)	5 (0.6)	3 (0.4)		
CCS class (n, %)						0.450	0.131
- 0	1,882 (60.0)	478 (60.1)	463 (57.5)	488 (61.0)	453 (61.4)		
- 1	781 (24.9)	199 (25.0)	210 (26.1)	185 (23.1)	187 (25.3)		
- 2	306 (9.7)	79 (9.9)	78 (9.7)	83 (10.4)	66 (8.9)		
- 3	68 (2.2)	18 (2.3)	25 (3.1)	13 (1.6)	12 (1.6)		
- 4	11 (0.4)	1 (0.1)	3 (0.4)	5 (0.6)	2 (0.3)		
- missing	13 (0.4)	2 (0.3)	3 (0.4)	5 (0.6)	3 (0.4)		
Bicuspid Aortic valve (n, %)	285 (9.1)	49 (6.2)	62 (7.7)	68 (8.5)	106 (14.4)	<0.001	**0.273**
Aortic stenosis (n, %)	2,299 (73.2)	609 (76.5)	621 (77.1)	577 (72.1)	492 (66.7)	<0.001	**0.235**
Aortic regurgitation (n, %)	398 (12.7)	100 (12.6)	89 (11.1)	107 (13.4)	102 (13.8)	0.370	0.084
LVEF %, median (IQR)	60 (55-65)	60 (55-66)	60 (55-65)	60 (55-65)	60 (55-65)	<0.001	0.200
Active endocarditis (n, %)	170 (5.4)	20 (2.5)	32 (4.0)	42 (5.3)	76 (10.3)	<0.001	**0.322**
Previous stroke (n, %)	96 (3.1)	24 (3.0)	22 (2.7)	22 (2.8)	28 (3.8)	0.594	0.060
Previous TIA (n, %)	62 (2.0)	14 (1.8)	18 (2.2)	12 (1.5)	18 (2.4)	0.526	0.068
Significant carotid artery disease (n, %)	14 (0.4)	4 (0.5)	2 (0.2)	3 (0.4)	5 (0.7)	0.627	0.063
Creatinine, mg/dl, median (IQR)	0.98 (0.80-1.10)	0.95 (0.79-1.09)	0.97 (0.80-1.12)	1.00 (0.82-1.10)	1.00 (0.82-1.08)	0.058	0.061
Chronic lung disease (n, %)	341 (10.9)	90 (11.3)	74 (9.2)	93 (11.6)	84 (11.4)	0.367	0.080

Previous cardiac surgery (n, %)	337 (10.7)	48 (6.0)	63 (7.8)	91 (11.4)	135 (18.3)	<0.001	**0.382**
EuroSCORE logistic, median (IQR)	6.23 (4.24-9.76)	6.61 (4.53-9.52)	6.34 (4.30-9.52)	6.38 (4.24-10.30)	5.75 (3.32-10.74)	0.012	0.166
EuroSCORE II, median (IQR)	1.84 (1.24-3.19)	1.89 (1.29-3.12)	1.74 (1.22-2.81)	1.96 (1.33-3.44)	1.77 (1.09-3.80)	0.020	**0.259**
Previous dialisys (n, %)	10 (0.3)	1 (0.1)	2 (0.2)	0 (0.0)	7 (0.9)	0.005	0.138
Urgency (n, %)	394 (12.6)	73 (9.2)	100 (12.4)	97 (12.1)	124 (16.8)	0.001	**0.226**
CPB minutes, median (IQR)	63.0 (49.0-79.0)	42.0 (37.0-46.0)	56.0 (52.0-60.0)	70.0 (65.5-75.0)	93.0 (84.0-106.0)	<0.001	

Values are n (%) unless otherwise designated as median and IQR (interquartile range). BMI: body mass index. CCS: The Canadian Cardiovascular Society Angina Score. CKD: Chronic Kidney Disease. CPB: cardiopulmonary bypass. EuroSCORE: european system for cardiac operative risk evaluation. LVEF: left ventricle ejection fraction. NYHA: New York Heart Association. TIA: transient ischemic attack
